# Microneedle-mediated transdermal bacteriophage delivery

**DOI:** 10.1016/j.ejps.2012.06.012

**Published:** 2012-09-29

**Authors:** Elizabeth Ryan, Martin J. Garland, Thakur Raghu Raj Singh, Eoin Bambury, John O’Dea, Katarzyna Migalska, Sean P. Gorman, Helen O. McCarthy, Brendan F. Gilmore, Ryan F. Donnelly

**Affiliations:** a School of Pharmacy, Queen’s University Belfast, Medical Biology Centre, 97 Lisburn Road, Belfast BT9 7BL, UK; b Crospon Ireland, Galway Business Park, Dangan, Galway, Ireland

**Keywords:** Bacteriophage therapy, Transdermal delivery, Hollow microneedle, Bacterial infection

## Abstract

Interest in bacteriophages as therapeutic agents has recently been reawakened. Parenteral delivery is the most routinely-employed method of administration. However, injection of phages has numerous disadvantages, such as the requirement of a health professional for administration and the possibility of cross-contamination. Transdermal delivery offers one potential means of overcoming many of these problems. The present study utilized a novel poly (carbonate) (PC) hollow microneedle (MN) device for the transdermal delivery of *Escherichia coli*-specific T4 bacteriophages both *in vitro* and *in vivo*. MN successfully achieved bacteriophage delivery *in vitro* across dermatomed and full thickness skin. A concentration of 2.67 × 10^6^ PFU/ml (plaque forming units per ml) was detected in the receiver compartment when delivered across dermatomed skin and 4.0 × 10^3^ PFU/ml was detected in the receiver compartment when delivered across full thickness skin. An *in vivo* study resulted in 4.13 × 10^3^ PFU/ml being detected in blood 30 min following initial MN-mediated phage administration. Clearance occurred rapidly, with phages being completely cleared from the systemic circulation within 24 h, which was expected in the absence of infection. We have shown here that MN-mediated delivery allows successful systemic phage absorption. Accordingly, bacteriophage-based therapeutics may now have an alternative route for systemic delivery. Once fully-investigated, this could lead to more widespread investigation of these interesting therapeutic viruses.

## Graphical abstract

**Figure:**
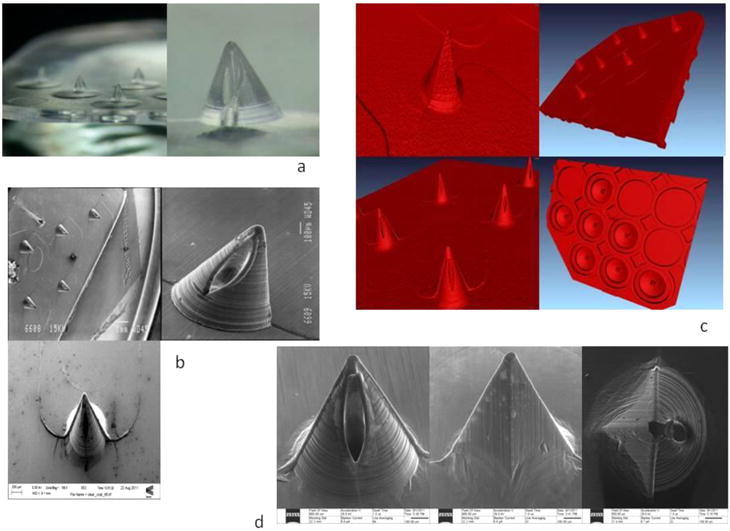

## 1 Introduction

Bacteriophages (20–200 nm in size) are bacterial viruses which specifically infect bacteria. In the case of lytic phages, they disrupt normal bacterial metabolism in favour of viral replication and cause the bacterium to rapidly lyse (Hendrix, 2002). Despite predating the discovery of antibiotics by several decades, bacteriophage therapy was largely supplanted by antibiotics and vaccines and their use in western medicine declined. However, the emergence of multidrug-resistant pathogenic bacteria, combined with a concomitant increase in numbers of immunosuppressed patients, raises concerns common to the ‘pre-antibiotic era’, which was characterised by untreatable infectious diseases. Whilst some new antibiotics have been developed, overall industry effort into antibacterial drug development has declined, with several major Pharma companies exiting the field or aggressively downsizing their development programmes (Payne and Tomasz, 2004). Therefore, development of alternative antimicrobial modalities is urgently required and has become a major priority in modern biotechnology (Sulakvelidze et al., 2001).

The possibility of utilising bacteriophage therapy to treat infectious diseases has received increasing attention in recent years, as several advantages over conventional therapeutic agents have been recognised. Bacteriophage are (i) highly specific, thus unlikely to disturb normal flora in the same manner as conventional chemotherapeutic agents and have the potential to be self-replicating, facilitating effective treatment by delivery of a low phage dose, and self-limiting therapeutic agents; (ii) safe, underscored by their extensive use in the former Soviet Union and Eastern Europe and their widespread sale in the US by companies such as Eli Lilly and E.R. Squibb & Sons in the 1930–1940s and (iii) are rapidly modifiable to combat emergence of bacterial resistance. Indeed, resistance may be easily circumvented by delivering a ‘phage cocktail’ directed against numerous strains of the target species. Significantly, phages are also capable of treating intra-cellular antibiotic-resistant pathogens, such as *Mycobacterium avium* and *Mycobacterium tuberculosis* (Broxmeyer et al., 2002).

Phage biology may be manipulated, primarily *via* phage display techniques, for a plethora of other applications in nanomedicine. Delivery of suitably-engineered phage has permitted isolation of allergens inducing IgE production using high throughput screening technologies (Rhyner et al., 2004). Gene delivery to mammalian cells has also been achieved by the use of single and double stranded phage by a number of groups (Yokohama-Kobayashi and Kato, 1993; Okyama and Berg, 1985; Larocca et al., 1999). This particular application may well have significant advantages over standard gene delivery vectors in terms of increased selectivity (and thus, efficacy) and reduced toxicity (Arap, 2005). Furthermore, tumour targeting peptides identified by phage display have been utilised for selective delivery of cytotoxic therapeutic agents to tumours, highlighting the potential for drug and drug delivery vector discovery by *in vivo* delivery of bacteriophage libraries (Arap et al., 1998). Phages can also be engineered to bear target-specific peptides or proteins for biorecognition, and thus may have application in development of novel chemical and biological sensors that may provide quantitative or semi-quantitative data through exploitation of a chemical or biological recognition element (Mao et al., 2009).

Bacteriophages do have some local activity when given orally, but only on infectious microorganisms in the gut. Absorption of intact bacteriophages into the systemic circulation does not take place following oral administration (Bruttin and Brüssow, 2004) and bile salts and intestinal carbohydrates may sequester the bivalent metal ions needed for phage replication (Chibani-Chennoufi et al., 2004). Inhalation-based delivery of bacteriophages has proved inefficient in animal studies (Huff et al., 2003). Consequently, parenteral delivery is the most routinely-employed method for administering bacteriophages. However, parenteral administration of therapeutics is associated with significant problems, including the need for trained personnel, the risk of blood-borne pathogen transmission, the frequent need for maintenance of an expensive ‘cold chain’ and relatively poor compliance (Morris et al., 1997). Nevertheless, despite the recognised problems with delivery and administration, there is increasing interest in development of phage-based therapeutics/diagnostics.

The success of bacteriophage-derived therapeutics and biosensors will ultimately rely on suitably robust, reproducible, delivery technologies. In particular, bacteriophage-based treatment of common infectious diseases will only be practical if these advanced medicines can be conveniently and routinely self-administered by patients in their own homes. Transdermal delivery (Williams, 2003; Barry, 2001) offers one potential means of overcoming many of the problems associated with systemic delivery of bacteriophages. Clearly bacteriophages, being viruses rather than small relatively lipophilic drug molecules, do not satisfy the criteria for efficient transdermal absoprtion. Nevertheless, the transdermal delivery of these potent therapeutic agents is of particular interest, as it may overcome many of the problems associated with conventional delivery methods. To date, transdermal delivery of bacteriophages has not been considered. However, novel microneedle technologies, developed by our Group and others, have now made this a possibility, particularly for thermolabile biomolecules and biological entities (Donnelly et al., 2010a, 2010b, 2011; Mikolajewska et al., 2010; Migalska et al., 2011; Prausnitz, 2004; Garland et al., 2011). In this paper, we report for the first time, design and evaluation of a novel hollow polymeric microneedle device for transdermal bacteriophage delivery.

## 2 Materials and methods

### 2.1 Materials

T4 bacteriophage ATCC® B11303 and host strain *Escherichia coli* 11303 ATCC® 11303 were purchased from LGC standards, Middlesex, UK. Luria Bertani (LB) agar was purchased from Sigma–Aldrich, Dorset, UK. Stock phage solutions were stored at 4 °C and protected from light. *E. coli* was frozen with cryoprotectant beads and glycerol and stored at −60 °C. Isoflurane inhalation anaesthetic was obtained from Abbott Laboratories Ltd., Kent, UK. All other chemicals used were of analytical reagent grade.

### 2.2 Fabrication of poly(carbonate) (PC) hollow microneedle arrays

Microneedles (MNs) were manufactured using a prototype micromoulding process. Mould cavities and inserts were micro-machined from brass and inset pins were machined from H-13 tool steel using a specialized Electric Discharge Machining (EDM) process. The moulds were run on an Arburg 221 KS Allrounder moulding machine. MNs were manufactured from PC. The prototype array of MNs consisted of seven needles at 3 mm centers on a 21 mm × 21 mm base. The MNs were 1 mm in height with a 100 μm off-centre through-hole. The aspect ratio was 1.6:1. The tip sharpness of the prototype needles was approximately 25 μm in radius. The MN array was ultrasonically welded to a reservoir array of the same material as the MN array consisting of a 5 μl reservoir well for each MN. A silicone sealing gasket was used in-between the MN array and reservoir array.

### 2.3 Microscopy techniques

To observe MN morphology, images of the MNs were taken using a Leica DC150 digital microscope (Leica, Wetzlar, Germany). MNs were attached to aluminium stubs using double-sided adhesive and coated at 2.5 kV, 18 mA with gold for 45 s (POLARON E5150, Gold Sputter Coater, Quorum Technologies, East Sussex, UK). The images were then obtained using a Scanning Electron Microscope (JOEL JSM 840, Joel Ltd., Tokyo, Japan).

The MN arrays were also visualised using a Phoenix X-ray nanotom system (GE, London, UK), under the following conditions; energy: 55 kV, current: 160 mA, nanotom mode: 0, voxel resolution: 10 μm, number of projections: 720, image averaging: 3, detector timing: 1500 ms, binning mode: 1 × 1 (no binning). The method involved the acquisition of a series of X-ray projection images at a known number of angular positions through 360°. Variation in the contrast of each projection image relates to how the x-rays are attenuated as they penetrate the sample. Axial slice views were computed from the X-ray projections using back projection reconstruction algorithms. 3D rendering of the all axial slice views allowed visualisation of the 3D model.

He-ion images of the MN arrays were generated using the Orion Helium- ion microscope (Carl Zeiss Smt GmBH, Oberkochen, Germany). He-ion technology relies on a novel high brightness helium ion source of atomic dimensions. The helium beam was focused on the sample with an ultimate probe size of 0.25 nm. The images provide rich surface–specific information due to the unique nature of the beam-sample interaction. The hollow MNs were imaged under the following conditions: Acceleration 29.0 kV, dwell time 1.0 μs, blanker current 6.7 pA, working distance 22–23 mm.

### 2.4 Insertion force experiments of PC MN arrays

The force required to successfully insert the PC MNs into excised porcine skin was determined using a TA.XT-plus Texture Analyser (Stable Micro Systems, Surrey, UK). Neonate porcine skin was obtained from stillborn piglets and immediately (<24 h after birth) excised and trimmed to a thickness of approximately 400 μm using an electric dermatome (Integra Life Sciences™, Padgett Instruments, NJ, USA). Skin was then stored in aluminium foil at −20 °C until further use but for no longer than 2 weeks. Skin was equilibrated in PBS for an hour and hair was removed using a disposable razor. The *SC* surface of the skin was dried with tissue paper and the skin was placed, dermis side down, on a 500 μm-thick sheet of dental wax, and this assembly was then secured on a wooden block for support. MNs were attached to the tip of a moveable cylindrical probe (length 5 cm, cross-sectional area 1.5 cm^2^) using cyanoacrylate adhesive (Loctite, Dublin, Ireland). The test station, in compression mode, then pressed MN arrays against the skin at a speed of 0.5 mm/s for 30 s with known forces of 0.05, 0.10 and 0.40 N/needle. Following MN removal, methylene blue solution (1% w/v) was applied onto the skin surface and left for 15 min. This solution was then gently wiped off, first with dry tissue paper and then with saline and alcohol swabs. The surface of the stained skin was then photographed using a digital camera (Nikon Coolpix I120®, Nikon UK Ltd., Surrey, UK) and the number of methylene blue stained micro-conduits was simply counted.

### 2.5 Optical coherence tomographic assessment of MN penetration into neonatal porcine skin

The penetration characteristics of MNs following insertion into excised full thickness (750 ± 20 μm) neonatal porcine skin was determined using optical coherence tomography (OCT). The skin was placed onto a section of dental wax for support. MNs were inserted using a custom-designed spring-activated applicator (Donnelly et al., 2010c), at a force of 11 N/per array, manually held in place and immediately viewed using an EX1301 OCT Microscope (Michelson Diagnostics Ltd., UK). The swept-source Fourier domain OCT system has a laser centre wavelength of 1305.0 ± 15.0 nm, facilitating real time high resolution imaging of the upper skin layers (7.5 μm lateral and 10.0 μm vertical resolution). The skin was scanned at a frame rate of up to 15 B-scans (2D cross-sectional scans) per second (scan width = 2.0 mm). Following MN removal, the microporated skin was immediately viewed using OCT, as above, to allow a determination of the depth and width of the pore created within the skin. 2D images were analysed using the National Institutes of Health imaging software ImageJ®. The scale of the image files obtained was 1.0 pixel = 4.2 μm, thus allowing accurate measurements of the depth of MN penetration and the width of pore created. The obtained 2D images were converted into a 3D representation using the rendering programme Voxx2. To allow easy differentiation between MN and skin layers, false colours were applied using Ability Photopaint® Version 4.14.

### 2.6 Fracture force experiments

**Fig. 1:**
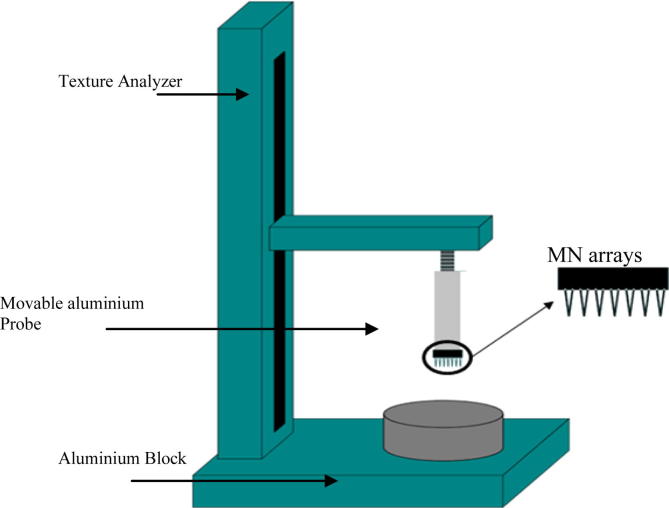
Illustration of texture-analyzer set up for determination of fracture forces of MN arrays.

In order to determine the axial forces (parallel to MN shaft) necessary for mechanical fracture of the MN, MNs were again fixed to the tip of the moveable cylindrical probe of the Texture Analyser using cyanoacrylate adhesive. An axial compression load was then applied. The test station pressed the MN arrays against a flat aluminium block at a rate 0.5 mm s^−1^ with defined forces for 30 s, as shown in Fig. 1. Pre-test and post-test speed was 1 mm s^−1^ and the trigger force was set at 0.049 N. MNs were subjected to defined forces of 0.05, 0.1, and 0.4 N/needle. All MNs of each array were visually examined using a digital microscope before and after fracture testing and changes in height were recorded by using the digital microscope’s computer software.

### 2.7 Production of hollow microneedle device

**Fig. 2:**
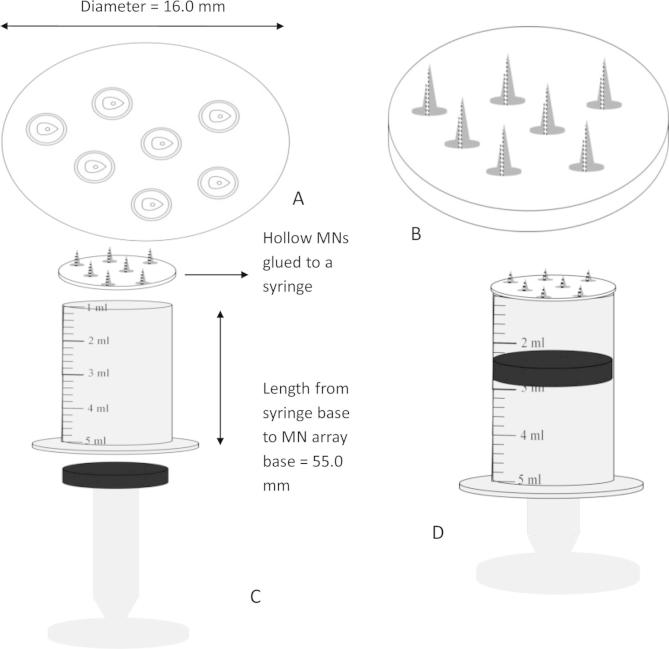
Assembly of hollow MN arrays showing (A) the top view of MNs (B) the lateral view of MNs (C) the three components of MNs, namely the MN array, the modified barrel and the rubber headed plunger (D) the MN arrays glued to the modified syringe.

The hollow MN device was manufactured by cutting off the tip of a 5 ml Terumo® syringe. The diameter of the syringe was 16.0 mm. The MN array was cut into a circular (diameter 16.0 mm) to fit directly onto the barrel of the syringe. It was sealed using a silicone membrane and the three parts were fixed together using cyanoacrylate glue (Loctite, Dublin, Ireland). Syringe base to MN array base measured 55.0 mm. The plunger of the syringe was not modified and measured 70.0 mm in length (Fig. 2).

### 2.8 Propagation of T4 phage to produce a high titre stock

An actively growing broth culture of the T4 phage host strain, *E. coli* 11303, was prepared 18–24 h prior to propagation of T4 phage culture. Plates of 1.2% LB agar plus 0.5% NaCl were pre-warmed in an incubator at 37 °C. The 0.6% LB agar (soft agar for overlay) (previously autoclaved) was liquefied in a water bath, then stored at 43–45 °C until required. One aliquot (60 μl) of the *E. coli* host strain was added to 2.5 ml of molten 0.6% LB agar. The surface of the plates were overlaid with the soft agar and allowed to set. Luria Bertani (LB) broth (1.0 ml) containing 0.5% NaCl was added to the vial containing freeze-dried phage and 0.1 ml of the rehydrated phage was spotted onto the overlay. The plate was tilted to spread the rehydrated phage over as much of the surface as possible. This was allowed to dry and incubated at 37 °C overnight.

After 24 h incubation, the soft agar was scraped from the surface of the agar plates using a sterile cell scraper. The soft agar was centrifuged at 1000 rpm for 25 min to sediment the cellular debris and agar. The supernatant was passed through a 0.22 μm Millipore filter and the filtrate was stored at 4–8 °C.

### 2.9 Enumeration of phage titre using the double layer plaque assay method

The double layer plaque assay method was adapted from a method devised by Adams (1959). An actively growing broth culture of *E. coli* 11303 was prepared 18–24 h before performing the plaque assay. Plates of 1.2% LB agar were pre-warmed in an incubator at 37 °C. Plates were prepared as previously described. Serial dilutions of the samples obtained from the *in vitro* release study were prepared from 10^−1^ to 10^−8^. The agar overlay was prepared by adding 60 μl of the *E. coli* innoculum into 3 ml of 0.6% top agar and poured immediately onto the 1.2% agar plates and agitated to ensure even distribution. Samples of each serial dilution (20 μl) were spotted onto the overlay, with 4–5 dilutions per plate. Each sample was spotted in triplicate to ensure reproducibility. The plates were incubated at 37 °C overnight. Plaques were subsequently enumerated on plates at each dilution. Plaques appear as defined, circular zones of clearance within the bacterial lawn, due to bacteriophage-mediated bacterial cell lysis. The concentration of bacteriophage present in each sample was calculated from the dilution in which plaques were most countable, and using the following equation:

(1) Number of plaques×dilution factor×50=Concentration in PFU/ml

where 20 μl is plated, ×50 to calculate PFU/ml. An average of the three results was taken as the phage concentration.

### 2.10 *In vitro* release studies of bacteriophage T4 stock using hollow MN device

The delivery of a stock solution (5 × 10^8^ PFU/ml) of T4 bacteriophage across neonatal porcine skin, using the hollow MN system was carried out using Franz diffusion cells (FDC-400 flat flange, 15 mm orifice diameter, mounted on an FDCD diffusion drive console providing synchronous stirring at 600 rpm and thermostated at 37 ± 1 °C, Crown Glass Co. Inc., Sommerville, NJ, USA). The orifice diameter in the receptor compartment was 15 mm. No donor compartment was used, to allow ease of use of the hollow MN device. The receptor compartment volume was calculated to be 12 ml. PBS pH 7.4 (11 ml) was accurately dispensed into the receptor compartment using a 5 ml Pipetteman®, assuming that the full 1000 μl would be delivered *via* the hollow MN device. The PBS was degassed prior to use by sonication. Compartment temperatures were kept constant at 37 °C by re-circulating water from a thermostatically controlled bath. The samples of dermatomed (400 μm) and full thickness (750 ± 20 μm) neonatal porcine skin were prepared by shaving carefully to remove hair and was pre-equilibrated in PBS pH 7.4 (PBS) for 1 h before beginning the experiments. A circular specimen of the skin was secured to the receptor compartment of the diffusion cell using cyanoacrylate glue (Loctite, Dublin, Ireland) with the *SC* side facing up. The hollow MN device, with air expelled, was carefully inserted into the fixed dermatomed skin sample and approximately 1000 μl was dispensed by exerting a constant pressure on the plunger of the assembled MN device. This was done in triplicate for both the dermatomed and full thickness skin. Using a long needle, 200 μl samples were removed from the side arm of the receptor compartment at defined time intervals and replaced with an equal volume of pre-warmed degassed PBS. The samples were assayed using the plaque assay method as described in Section 2.9.

### 2.11 *In vivo* delivery of bacteriophage T4 via the hollow microneedle device

Four male Sprague–Dawley rats weighing 336 ± 14 g were used in the experiment. To prevent hair from interfering with dermal contact of the MN system, animals were anaesthetised using gas anaesthesia (2–4% Isoflurane in oxygen). Before the experiment, the hair was removed with an animal hair clipper. Additionally, depilatory cream (Boots Expert®, The Boots Company PLC, Nottingham, UK) was used to remove any residual hair. Skin barrier function was confirmed as intact on a case by case basis by standard transepidermal water loss measurements (Delfin Vapometer®, Delfin Technologies Ltd., Paris, France). A bacteriophage stock of concentration 4 × 10^9^ PFU/ml was used in the experiment. A volume of approximately 250 μl was administered at four different sites on the back of each rat. Rats were anaesthetized prior to administration of phages through the hollow MN system. The phage was delivered by manually pushing the barrel of the device into the rat skin until the hollow MN device was firmly in place and accurately pipetting 250 μl into the barrel. The plunger was then carefully pressed downwards through the barrel and held for 30 s. After phage administration, blood samples (100 μl) were collected at different time points over a 24 h period by lateral tail vein prick. Samples were taken at 0.5 h, 1 h, 1.5 h, 2 h, 4 h, 6 h and 24 h. All animal experiments were conducted with ethical approval according to EC Directive 86/609/EEC. The MN Research Group at Queen’s is committed to the three “R” principles of animal testing i.e. replacement–substituting alternative non-animal systems in place of live animal testing, reduction–using the fewest number of animals possible and refinement–developing procedures that limit the potential for discomfort to animals.

### 2.12 Blood calibration curve

A calibration curve of known phage concentration within rat blood versus detectable phage concentration was constructed. A phage stock of 3 × 10^8^ PFU/ml was used in the experiment. A 20 μl aliquot of this phage stock was added to 180 μl of rat blood (i.e. a 1 in 10 dilution) and 20 μl of this dilution was added to another 180 μl of rat blood. This serial dilution was continued to an expected 3 PFU/ml concentration. Plaque assays were carried out in triplicate and the average PFU/ml ± S.D. was plotted *via* the concentration calculated from phage stock. This curve was used to correlate the actual phage stock concentration to concentrations detected from blood samples. Linear regression analysis was used to construct the equation of the line. The correlation coefficient (*R*^2^) was also calculated to assess the linearity of the data.

### 2.13 Statistical analysis

Where appropriate, statistical analyses of the results were performed with a one-way analysis of variance, and a two-way analysis of variance (ANOVA). In all cases *p *< 0.05 was taken to represent a statistically significant difference. The software package used was GraphPad Prism 5 (GraphPad software Inc., San Diego, California, USA).

## 3 Results

**Fig. 3:**
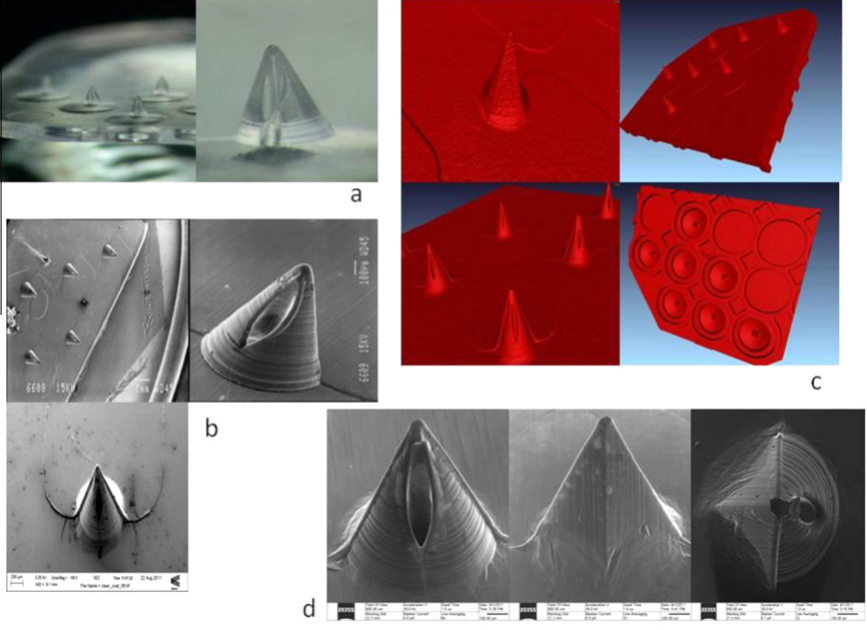
Images of PC hollow MN arrays using a variety of microscopy techniques. (a) Digital microscope images of single MN and a section of the MN array. (b) SEM images of a single MN, the bore of the MN and a section of the MN array. (c) X-ray microtomography images of single MNs, the bore of the MN and the Mn array. (d) He-ion images of a single MN, illustrating the bore of the needle and a radial view of the MN.

The images of the PC MN arrays are presented in Fig. 3. The mean height and base diameter for the PC MNs were approximately 995 μm and 750 μm, respectively. The hollow bore diameter was ≈100 μm. The aspect ratio was 1.3. The X-ray tomography images illustrate both the MN array and also the structure of the reservoirs at the base of each MN. The He-ion technology produced ultra sharp images of the PC needles. The rich surface specific information is due to the unique nature of the beam- sample interaction.

From the insertion forces studies of the PC arrays prior to fabrication of the MN device, it was observed that, at all three forces investigated (i.e. 0.05, 0.1 and 0.4 N/needle), MNs penetrated the *SC* of the skin. Therefore, 100% penetration efficiency was observed, regardless of the applied force.

Light microscope analysis showed that no decrease in MN height was observed upon removal from skin, regardless of the force of application.

**Fig. 4:**
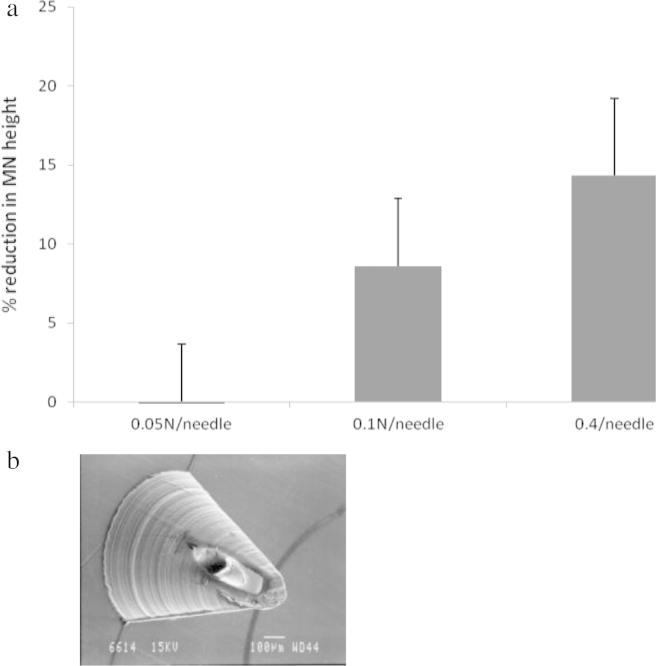
(a) Influence of applied force on the reduction of PC MN height. Means ± S.D. *n* = 7 MN on a single array (b) SEM image of following 0.4 N/needle axial force application on PC MNs.

**Fig. 5:**
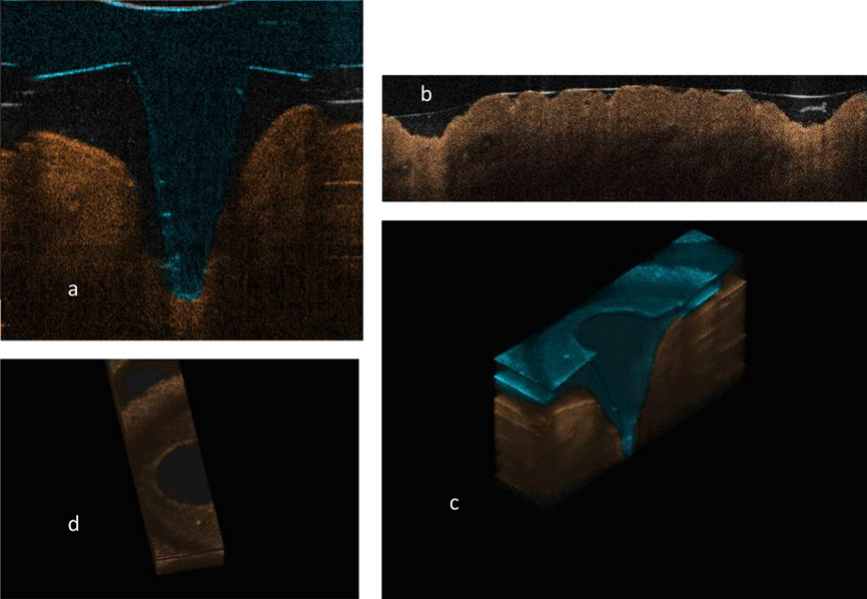
OCT images of PC MNs within skin. (a) A 2D OCT image of MNs *in situ*, following insertion into neonatal porcine skin at an application force of 11.0 N. (b) 2D image of the pores created within neonatal porcine skin, following the insertion and removal of MNs. (c) A 3D representation of MNs *in situ*, following insertion into neonatal porcine skin at an application force of 11.0 N. (d) 3D top view image of the pores created within neonatal porcine skin, following the insertion and removal of MNs.

**Fig. 6:**
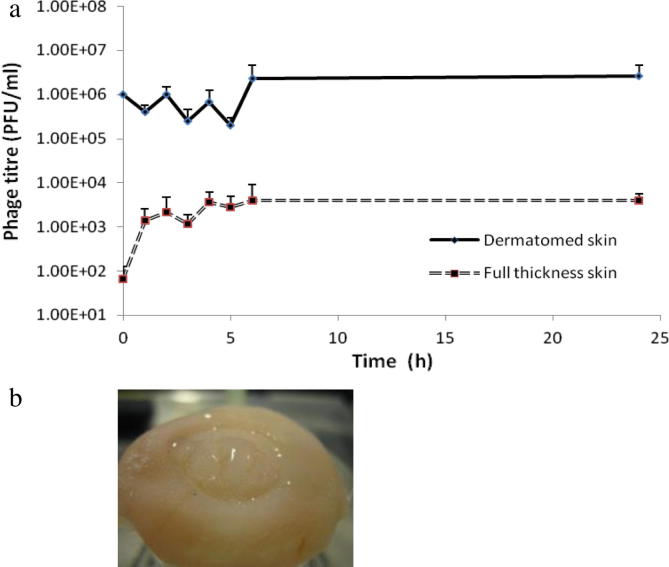
(a) Release study results of 1 ml phage stock delivered through dermatomed and full thickness skin using the hollow MN device. Means ± S.D., *n* = 3. (b) Photographic evidence of the “pool” created on top of full thickness skin following delivery using the hollow MN device.

Fracture force studies carried out on the MNs can be observed in Fig. 4a. At forces of 0.05 N/needle, there was no significant change in MN height. However, when the axial force was increased, the% reduction in height increased. Fig. 4b shows the morphology of MNs following 0.4 N/needle force application, with apparent damage at the tip of the needles. The 2D OCT image of the MNs following insertion into neonatal porcine skin is illustrated in Fig. 5. It was found that the MNs penetrated to an approximate depth of 700 μm and created a pore of approximate width 600 μm whilst the MNs were *in situ*. Fig. 5 also shows a 3D image of MNs *in situ* following insertion into neonatal porcine skin. It was found that, immediately following the removal of MNs from the neonatal porcine skin, the residual skin pore had a depth of approximately 210 μm, and a width of approximately 600 μm but quickly closed over (1 h, data not shown). 2D and 3D images of residual pores in the neonatal porcine skin immediately following the removal of the MNs are also shown. Release studies through dermatomed skin showed that the hollow MN device had the ability to fully penetrate the dermatomed skin (as was observed) and deliver bacteriophage transdermally. There was a small amount of liquid remaining on the surface of the skin following application. Accordingly, 100% delivery was not expected. A 1 ml volume of a 5 × 10^8^ PFU/ml stock was delivered into 11 ml PBS in the Franz cell donor compartment. Therefore, 4.5 × 10^7^ PFU/ml would be the maximum phage amount to be detected if 100% delivery occurred. Thirty minutes following delivery, 1 × 10^6^ PFU/ml was detected within the receptor compartment, as determined by plaque assay (Fig. 6a). Amounts of phage detected stayed within 1 × 10^6^ ± 1 log up to the 24 h time point. This is regarded as a constant level, as variability of this kind is common with plaque assay results (Darling et al., 1998).

Delivery of stock solution through full thickness skin proved difficult. MNs did not penetrate all layers of the skin and the resistance provided by the dermal layer meant that solution flow was reduced, yielding a pool of liquid the skin surface (Fig. 6b).

**Fig. 7:**
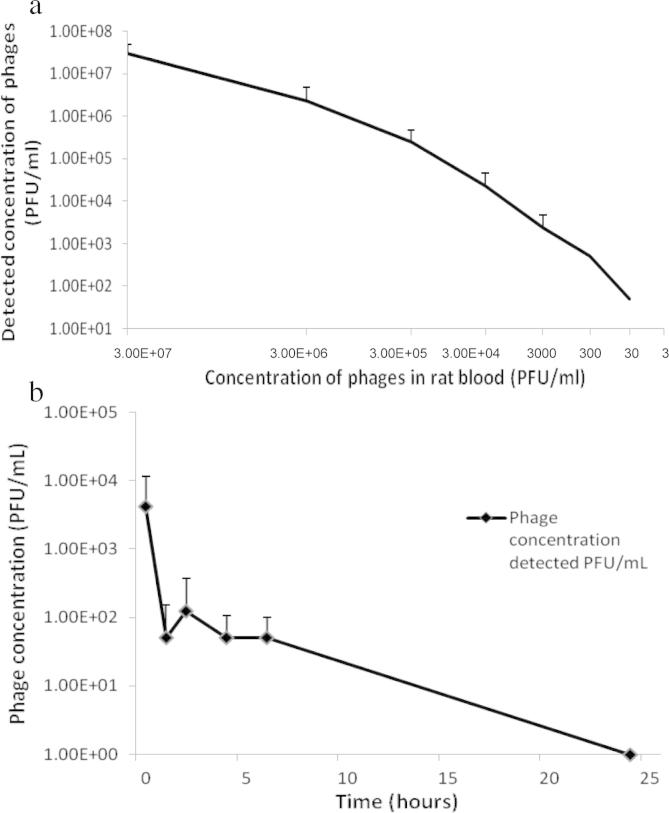
(a) Calibration curve of phages in rat blood versus detectable phages via plaque assay, illustrating the linearity of plaque assay results taken from the *in vivo* study, with a comparable plaque assay of phage in rat blood. Means ± S.D., *n* = 3. (b) *In vivo* release study results of T4 bacteriophage delivered by hollow PC MNs.

**Fig. 8:**
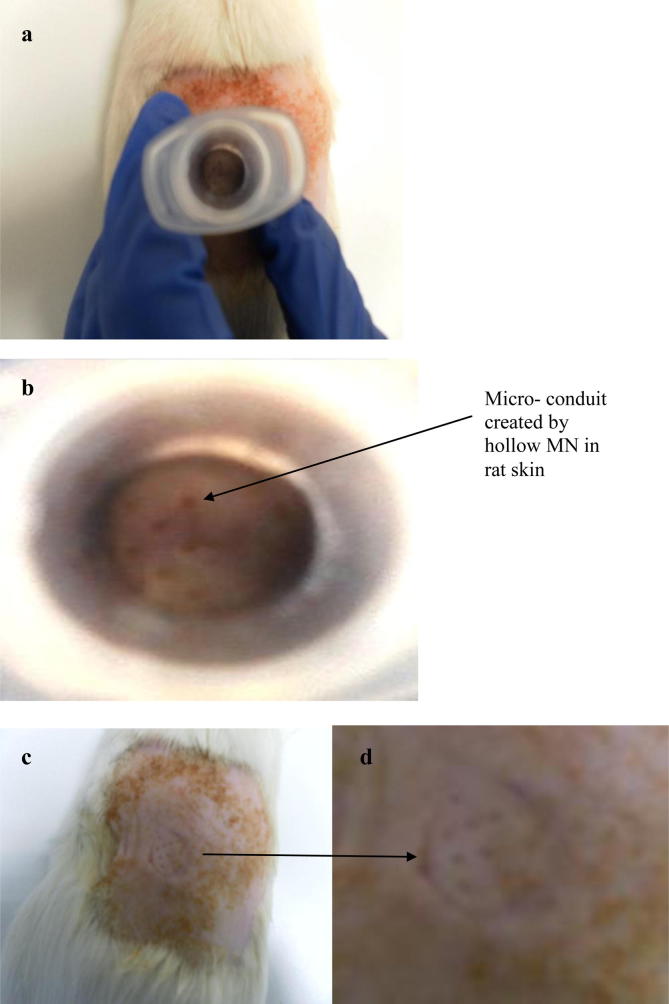
(a) Image of hollow MN device inserted through the skin of a rat, prior to administration of phage stock. (b) Close up of microporations through the rat skin created by hollow MNs (c) Image of microporations created by the hollow MN device in one of the rats, following administration of phage stock. (d) Close up of microporations through the rat skin created by hollow MNs, following phage stock administration.

The calibration curve (*R*^2^ = 0.992) constructed showed that phages were detectable in rat blood to a concentration of 30 PFU/ml (Fig. 7a). Phage concentrations detected at each timepoint are presented in Fig. 7b. Phage was detected at a concentration of approximately 4 × 10^3^ PFU/ml 30 min after phage administration. This phage concentration reduced rapidly at the next time point with an average 50 PFU/ml at 1.5 h and 125 PFU/ml at 2 h. Hypothetically, 1 ml of a 4 × 10^9^ PFU/ml stock was administered to each rat (although it is known that 100% delivery did not occur due to backflow of phage stock – Fig. 8). These results suggest that phages were successfully delivered into the systemic circulation. However, phages were also cleared quickly from the system, with an over 2 log reduction in phage concentration from 30 min to the 1 h time point. No phage was detected at the 24 h time point (Fig. 7b). The variation in plaque assay results from the 1 h to the 6 h time points can be explained by the known inherent variation of the microbiological plaque assay itself, as outlined above.

## 4 Discussion

A recent review by our Group illustrated the need for more diverse delivery systems to improve the breath of phage therapy applications (Ryan et al., 2011).The present study successfully delivered viable T4 bacteriophage transdermally both *in vitro* and *in vivo* using a novel hollow MN system. MN–mediated transdermal delivery punctures the skin and by-passes the *SC* to create transient aqueous transport pathways of micron dimensions. This, in turn, enhances transdermal permeability (Tanner and Marks, 2008). MNs possess many advantageous attributes including painless delivery, simple and affordable fabrication and the elimination of the threat of cross-contamination that parenteral delivery poses (Donnelly et al., 2010b, 2011; Garland et al., 2011). In dermatomed skin, it was found that holes could be detected in porcine skin at 0.05 N/needle and 0.1 N/needle. This confirmed that the *SC* barrier would be penetrated at each of these insertion forces. As the *SC* barrier is the principal barrier of the skin, once this barrier is breached, then transdermal transport is solely controlled by the properties of the drug delivery device employed, rather than the *SC*, as the viable epidermis does not constitute a meaningful barrier to drug penetration (Tanner and Marks, 2008).

Once it was confirmed that rat blood did not interfere with the plaque assay, a calibration plot was carried out to assess what concentration of phages could be detected using the assay. With a starting concentration of 3 × 10^8^ PFU/ml, it was found that the minimum limit of detection for the assay was 30 PFU/ml. The reduced concentration detected from the full thickness skin experiment (approximately 3 log) compared to dermatomed skin was due in part to the accumulation of phage stock on the surface of the skin during phage delivery into full thickness skin. As MN could not penetrate fully through full thickness skin, there was a high amount of pressure which pushed the liquid to the surface of the skin instead of through the skin. Therefore, it was expected that the results for full thickness skin would be lower than for dermatomed skin. Examples of how clogging of the needle bore opening during MN insertion and MN flow resistance due to dense dermal tissue compressed around the MN tip has previously been described (Gardeniers et al., 2003; Martanto et al., 2006).

To combat the problem of phage stock loss on the surface of the skin, a slightly altered administration procedure was adopted for the *in vivo* study. Instead of a single administration at one site of 1 ml phage stock, four 250 μl aliquots were administered at four different sites as it was hoped that a reduction in volume at each site, would allow an increase in the volume of stock delivered through the skin. The observed phage plasma profile suggests that this indeed was the case. Indeed, the *in vivo* study proved, for the first time, that live virus particles can be delivered transdermally through a MN system. A previous study carried out by Inchley (Inchley, 1969) reported that T4 bacteriophage administered to mice by intravenous injection (5 × 10^8^ PFU in 0.1 ml saline) were rapidly cleared from the systemic circulation by the Reticuloendothelial system (RES). It was found that the majority of phage (more than 99%) was phagocytosed during the first 30 min. Clearance continued at this rate up to 1 h, after which a prolonged phase of slower elimination occurred. By 6 h, approximately 10^4^ PFU/ml blood could be recovered and it had reduced to 2.7 × 10^2^ PFU/ml by 48 h. The study also concluded that 70–90% of recovered activity was located in the liver.

The present *in vivo* study detected 4.13 × 10^3^ PFU/ml 30 min following delivery. The absence of a suitable bacterial infection, which would have allowed the phages to replicate, meant that phages were cleared rapidly, as described above. It should also be pointed out that, if the original concentration of phage stock could be increased to 10^12^–10^13^ PFU/ml, a phage concentration of approx 10^7^ could possibly be achievable using the hollow MN device. Some recent studies have examined the effect of phage concentration on the success of phage therapy. Barrow and co-workers (Barrow et al., 1998) reported intramuscular administration of bacteriophage R could control *E. coli* septicaemia in chickens and meningitis in calves, and that a concentrations of phage as low as 10^2^ PFU intramuscularly provided some protection against *E. coli* K1^+^ induced mortality (mortality 2/5 animals), however this protection was not statistically significant. In this study, higher concentrations (10^4^ and 10^6^ PFU administered intramuscularly provided significant protection to both newly hatched and 3 week old chickens (zero mortality). Generally, *in vivo* phage therapy studies administered *via* the parenteral route require phage concentrations of 10^7^–10^10^ PFU/ml for full eradication of bacterial infections. This depends on the concentration of each bacterial species within the body (Biswas et al., 2002; Cerveny et al., 2002; Matsuzaki et al., 2003; Wills et al., 2005; McVay et al., 2007; Capparelli et al., 2007). As has been explored by Payne et al. (2000) and Payne and Jansen (2003), each phage-bacteria relationship is unique, the concentrations of phage needed to eradicate specific concentrations of bacteria need to be characterised independently. Capparelli et al. (2007) completed a study in which *S. aureus* systemic infections were challenged intravenously with phage M^SA^. A control group was set up in which 10^8^ CFU/mouse of *S. aureus* A170 was injected intravenously. Three other groups were intravenously treated with phage M^SA^ at final concentrations of 10^7^, 10^8^ and 10^9^ PFU/mouse respectively. All mice in the control group and the lowest titre group (10^7^) died within 4 days. The survival rate 10^8^ group was 40% and the mice treated with the highest concentration (10^9^) all survived. This example shows how each phage-bacteria relationship has a concentration threshold at which phage therapy will be successful and therefore a general statement cannot be made. If more phage was required, more MN-based “injections” could simply be made.

This hollow MN device successfully delivered a stock of T4 bacteriophage both *in vitro* and *in vivo*. Clearance occurred rapidly in the *in vivo* rat models, as expected, due to the lack of an infection model. It would be useful, in future studies, to carry out a similar experiment using an *E. coli* rat infection model to demonstrate the effectiveness of the MN-delivered phage in eradicating infections and to study the replication of phages and pharmacokinetics of the phage-bacteria system.

This is the first time that bacteriophages have been successfully delivered transdermally. Bacteriophage delivery has the potential to effectively improve the treatment of bacterial infections. It could be a suitable alternative to antibiotic therapy in some cases and may help overcome the present problem of antibiotic bacterial resistance. Advantages of bacteriophages for treatment of bacterial infections include their high specificity, self replication and good safety profiles. Aside from antibacterial therapy, phages have a plethora of other exciting applications. The possibility of delivering phages *via* an easy to use MN device removes the risks associated with parenteral delivery and would possibly allow for patient self-administration. In order to achieve this, however, extensive further studies are required in terms of delivery device optimisation and, ultimately, human clinical trials.
